# Elevated glucose levels in melanoma patients − a real-world analysis^☆^

**DOI:** 10.1016/j.jcte.2025.100405

**Published:** 2025-06-26

**Authors:** Joan Walter, Bojan Bojanic, Manuel Dittli, Nadia Fehr, Thomas Sartoretti, Moritz Schwyzer, Katharina Binz, Antonio G. Gennari, Matthias Ernst, Martin W. Huellner, Michael Messerli

**Affiliations:** a Department of Endocrinology, Diabetology and Clinical Nutrition, University Hospital Zurich, Zurich, Switzerland; b Department of Nuclear Medicine, University Hospital Zurich, Zurich, Switzerland; c Curana Endocrinology, Diabetology and Obesity Zurich, Hofwiesenstrasse 350, Zurich, Switzerland; d University of Zurich, Zurich, Switzerland; e Institute of Food, Nutrition and Health, Health Sciences and Technology, ETH Zurich, Zurich, Switzerland

**Keywords:** Melanoma, Diabetes, Capillary fasting blood glucose, Screening

## Abstract

**Aim:**

To assess the glycemic status of consecutive melanoma patients undergoing standardized capillary fasting blood glucose (cFBG) assessment prior to fluorodeoxyglucose positron emission tomography/computed tomography (FDG PET/CT) examination.

**Methods:**

This retrospective study included 336 consecutive melanoma patients at the University Hospital Zurich, Switzerland. Fasting cFBG levels were measured prior to FDG PET/CT and classified according to American Diabetes Association guidelines. Multivariable linear regression analysis was performed to identify independent predictors of cFBG levels. Sensitivity analyses were performed on patients examined before 11 AM and fasting for 8 h as well as patients without known diabetes.

**Results:**

The cohort included 336 melanoma patients with a median age of 67 years (IQR 57–76), 36 % female (122/336), and 12 % (40/336) with known diabetes mellitus. The median cFBG was 103 mg/dL (IQR 94–112; 5.7 mmol/L, IQR 5.2–6.2). Overall, 58 % (194/336) of patients had non-normal cFBG levels (≥100 mg/dL; ≥5.6 mmol/L), consistent with findings from a sensitivity analysis of patients presenting before 11 AM, where 58 % (115/198) exhibited non-normal levels. Excluding patients with known diabetes, 56 % (165/296) of patients had non-normal cFBG levels, with 7 % (20/210) having levels ≥126 mg/dL (≥7.0 mmol/L), indicative of possible undiagnosed diabetes mellitus. Multivariable linear regression analysis identified male gender, active disease, and subcutaneous fat as independent predictors of cFBG levels, whereas traditional risk factors such as BMI, visceral fat, hypertension or lack of exercise were not independent predictors.

**Conclusion:**

More than half of melanoma patients have elevated cFBG levels, even in those without known diabetes, highlighting the need for improved glycemic screening and management.

## Introduction

Cancer patients are at increased risk of developing new onset diabetes mellitus (DM) or hyperglycemia irrespective of a preexisting diagnosis of diabetes [1,2]. It is estimated that about 20 % of cancer patients concurrently have diabetes mellitus likely due to overlapping risk factors between the conditions [2]. Furthermore, next to corticosteroids, several of the newer cancer treatments can worsen glycemic control with or without known diabetes and trigger new onset of diabetes including autoimmune DM [3]. During chemotherapy, it is estimated that hyperglycemia affects approximately 10 %–30 % of cancer patients [2]. In melanoma patients, the presence of diabetes has been associated with poorer survival outcomes and an increased risk of metastasis [4]. Worsening of glycemic control and hyperglycemia in particular presents significant challenges in cancer patient care. First, several studies have shown that compared to normal-glycemic patients, hyperglycemia was independently associated with worse overall survival and risk of cancer recurrence in both solid and hematological cancers [5,6]. Second, although mostly based on observational studies, there is data that suggests that hyperglycemia may directly impair the effectiveness of chemotherapy [7]. Third, poor glycemic control in cancer patients with and without diabetes is associated with increased risk of toxicities such as chemotherapy induced neutropenia, increased risk of infection and may cause early treatment reductions or cessation [8]. Fourth, hyperglycemia and its symptoms can be debilitation especially together with anti-cancer therapy side effects and data shows that patient reported outcomes is significantly worse in patients with both cancer and diabetes as compared with only either disease [9].

Therefore, the aim of this study is to screen the glycemic status of unselected, consecutive melanoma patients at different stages of care − diagnosis, ongoing therapy, and post-treatment follow-up − utilizing the standardized fasting blood glucose (FBG) assessment at the nuclear medicine department.

## Material and methods

The study was conducted at the University Hospital Zurich, Switzerland, which hosts the region's largest nuclear medicine department. It received approval from the local ethics committee (Kantonale Ethikkomission Zürich, Zurich, Switzerland, Basec-Nr.: 2023-02276) and was carried out in accordance with the Declaration of Helsinki and relevant guidelines and regulations. Written informed consent for the scientific use of medical data was obtained from all patients.

### Study population

This retrospective study included consecutive melanoma cancer patients (identified by ICD C43 code) undergoing clinically indicated oncologic fluorine-18 fluorodeoxyglucose positron emission tomography/computed tomography (F18-FDG PET/CT) between June 2023 and February 2024. Clinical information was used as recorder in the hospital clinical information system and provided by the patient during examination using a standardized questionnaire.

### Capillary fasting blood glucose measurements and definitions

As part of routine clinical practice, patients were instructed to fast before their examination, and patients scheduled in the afternoon were instructed to fast for at least 4 h before the examination. Patients with morning examinations were assumed to be fasting since their last meal the previous day. Capillary FBG was measured after confirming that the patient was fasting for at least 4 h by trained nurses using Accu-Chek® Inform II device (Roche Diagnostics, Switzerland). The Accu-Chek® Inform II demonstrated excellent correlation with clinical laboratory glucose measurements (concordance correlation coefficient = 0.95, intraclass correlation coefficient = 0.96), with deviations, when present, being falsely low [10]. For this study capillary FBG levels were defined according to the American Diabetes Association: Normal FBG, <100 mg/dL (<5.6 mmol/l), prediabetic FBG, 100–125 mg/dL (5.6–6.9 mmol/l) and diabetic FBG, ≥126 mg/dL (≥7.0 mmol/l). Additionally, the subgroup of patients with capillary glucose levels ≥200 mg/dL (≥11.1 mmol/L, equivalent to diabetic random glucose level) was evaluated. Importantly, for the formal diagnosis of diabetes, current guidelines recommend the measurement of fasting plasma glucose using venous plasma samples after at least 8 h of fasting [11]. Visceral and subcutaneous fat were measured standardized and CT-based as the distance between the internal surface of the abdominal muscle and the anterior wall of the aorta (at the level of the bifurcation) and the distance from the outer layer of the abdominal muscle to the cutis measured at the same level [12].

### Statistical analysis

Comparisons of baseline characteristics were performed using the Mann Whitney-*U* test, Kruskal-Wallis and Fisher s exact test. Continuous variables are presented as median and respective interquartile range (IQR) and categorical variables are presented as frequencies and respective percentages. Confidence intervals (CI) of proportions are exact binomial 95 % CIs. Spearman's rank correlation was utilized to analyze the relationships between variables. Linear regression analysis was conducted to identify independent predictors of capillary FBG in multivariate analysis. Variables were included in multivariable analysis if their univariate coefficient had a p-value < 0.2. Sensitivity analyses of patients that had their examination before 11 AM in the morning and were likely fasting for at least 8 h as well as excluding patients with known diabetes were performed. Statistical analyses were performed with R version. All hypothesis testing was two-tailed, and a p-value < 0.05 was considered statistically significant.

## Results

Overall, 336 consecutive melanoma patients that provided written informed consent were included (Table 1). The median age of the included patients was 67 years (IQR 57–76), 36 % (122/336) were female and 12 % (40/336) had a known diabetes mellitus.

**Table 1:** Patient characteristics stratified by capillary fasting glucose levels.

	Overall, N = 336	<100 mg/dL (<5.6 mmol/l), N = 142, 42 %	100–125 mg/dL (5.6–6.9 mmol/l), N = 159, 47 %	≥126 mg/dL (≥ 7.0 mmol/l), N = 35, 10 %	p-value
**Capillary FBG [IQR],**					<0.001
mg/dL	103 [94, 112]	92 [86, 95]	106 [104, 113]	137 [131, 151]	
mmol/l	5.7 [5.2, 6.2]	5.1 [4.8, 5.3]	5.9 [5.8, 6.3]	7.6 [7.3, 8.4]	
**Female, %**	122 (36 %)	58 (41 %)	55 (35 %)	9 (26 %)	0.534
**Age [IQR], years**	67 [57, 76]	67 [55, 76]	66 [58, 77]	75 [64, 81]	0.063
**BMI [IQR], kg/m^2^**	25 [23, 28]	25 [23, 26]	26 [24, 29]	27 [23, 29]	0.009
<18	9 (3 %)	5 (4 %)	2 (1 %)	2 (6 %)	
18–25	136 (41 %)	65 (46 %)	61 (38 %)	10 (29 %)	
25–30	137 (41 %)	59 (42 %)	62 (39 %)	16 (46 %)	
30–35	39 (12 %)	6 (4 %)	27 (17 %)	6 (17 %)	
35–40	13 (4 %)	6 (4 %)	6 (4 %)	1 (3 %)	
>40	2 (1 %)	1 (1 %)	1 (1 %)	0 (0 %)	
**Ever smoker, %**	155 (46 %)	65 (46 %)	70 (44 %)	20 (57 %)	0.368
**Known diabetes mellitus, %**	40 (12 %)	11 (8 %)	14 (9 %)	15 (43 %)	<0.001
**Hypertension, %**	128 (38 %)	48 (34 %)	60 (38 %)	20 (57 %)	0.039
**Regular exercise, %**	204 (61 %)	91 (64 %)	102 (64 %)	11 (31 %)	0.001
**Active melanoma, %**	99 (30 %)	35 (25 %)	49 (31 %)	15 (43 %)	0.093
**Subcutaneous fat [IQR], mm**	22 [17, 29]	21 [18, 27]	22 [19, 30]	21 [16, 29]	0.224
**Visceral fat (IQR), mm**	53 [39, 75]	49 [36, 61]	56 [44, 79]	65 [47, 89]	0.001

FBG = fasting blood glucose; BMI = body mass index; IQR = interquartile range.

The median capillary FBG was 103 mg/dL (IQR 94–113; 5.7 mmol/L, IQR 5.2–6.3) and overall, 58 % (194/336) of patients had a non-normal capillary FBG of ≥100 mg/dL (≥5.6 mmol/L) with 10 % (35/336) having capillary FBG values ≥126 mg/dL (≥7.0 mmol/L) and 1.2 % (4/336) having capillary FBG values ≥200 mg/dL (≥11.1 mmol/L). This was confirmed separately in a sensitivity analysis of the 198 patients with an early examination (before 11 AM) and fasting since the previous day presenting with a median capillary FBG of 103 mg/dL (IQR 94–112; 5.7 mmol/L, IQR 5.2–6.2) a non-normal capillary FBG ≥ 100 mg/dL (≥5.6 mmol/L) in 58 % (115/198) of patients, a capillary FBG ≥ 126 mg/dL (≥7.0 mmol/L) in 11 % (22/198) of patients and a capillary FBG ≥ 200 mg/dL (≥11.1 mmol/L) in 2 % (1/198) of patients (*p* = 0.832 for comparison median capillary FBG between early examination and later examination).

Excluding 40 patients with known diabetes mellitus, median capillary FBG was 103 mg/dL (IQR 94–110; 5.7 mmol/L, IQR 5.2–6.2 mmol/L) and overall, 56 % (165/296) of patients had a non-normal capillary FBG of ≥ 100 mg/dL (≥5.6 mmol/L) and capillary FBG ≥ 126 mg/dL (≥7.0 mmol/L) in 7 % (20/296) of patients.

Multivariate linear regression analysis identified male gender (−5.1, 95 % CI −9.1 to −1.0, *p* = 0.014), active disease (5.8, 95 % CI 1.6 to 10.0, *p* = 0.007), and subcutaneous fat (6.1, 95 % CI 0.8 to 11.5, *p* = 0.024) as independent predictors of capillary FBG in multivariate analysis, whereas traditional risk factors such as BMI (−1.3, 95 % CI −19.2 to 16.6, *p* = 0.887), visceral fat (4.6, 95 % CI −0.9 to 10.1, *p* = 0.103), hypertension (3.3, 95 % CI −0.8 to 7.3, p = 0.114), or lack of exercise (−2.8, 95 % CI −6.8 to 1.2, *p* = 0.164) were not independent predictors of capillary FBG.

## Discussion

This study assessed the glycemic status in consecutive melanoma patients that presented for standardized capillary FBG assessment prior to FDG PET/CT examination. We report three major results. First, nearly 2 out of 3 melanoma patients presenting for FDG PET/CT examination exhibited non-normal capillary FBG levels. This finding was consistent in a sensitivity analysis conducted on patients presenting early in the morning fasting since the previous day. Second, even after excluding patients with known diabetes, more than half (56 %) of melanoma patients without known diabetes undergoing FDG PET/CT examination had non-normal capillary FBG levels. Additionally, 7 % of these patients had capillary FBG measurements of ≥126 mg/dL (≥7.0 mmol/L), indicative of possible undiagnosed diabetes mellitus. Third, multivariable linear regression analysis showed that subcutaneous fat rather than BMI or visceral fat and male sex was independently associated with these levels. This association may reflect a loss of the protective, buffering role of subcutaneous fat in glucose homeostasis, as impaired expandability can promote ectopic lipid deposition and insulin resistance even in the absence of excess visceral fat [13]. Additionally, traditional risk factors such as lack of exercise, hypertension or smoking status were predictors of higher blood sugar levels.

This corroborates previous studies and confirms the high prevalence of non-optimal glycemic control in melanoma patients in a real-world setting. Research indicates that hyperglycemia is associated with poorer outcomes in cancer patients, including reduced overall survival and increased cancer recurrence across various types of cancers such as melanoma, and particularly can impact disease-free and overall survival [6,7]. Hyperglycemia may also compromise chemotherapy effectiveness, increase toxicity risks such as neutropenia and infections, and exacerbate the debilitating effects of treatment, leading to poorer patient-reported outcomes in those with both cancer and diabetes [[7], [8], [9]]. For melanoma specifically, insulin resistance has been identified as potential risk factor and the presence of diabetes has been associated with poorer survival outcomes and an increased risk of metastasis [4,14]. The sparse existing real-world data indicates that cancer patients with hyperglycemia experience higher rates of emergency hospital admissions compared to those without [2,15]. Early recognition and management of glycemic levels could likely mitigate this risk [2]. Additionally, elevated glucose levels as measured in this study and even mild hyperglycemia can directly compete with FDG, reducing tumor-to-background contrast and compromising standardized uptake value measurements, thereby affecting diagnostic accuracy [16,17].

Some limitations of this study should be considered when interpreting these findings. This was a retrospective analysis of consecutive patients presenting to a large tertiary hospital, inherently limiting causal inference. Despite being a high-volume site with most patients providing written informed consent, prospective studies are necessary to validate the results. The analyses were based on a single capillary glucose measurement. Although the testing was conducted by trained personnel and calibrated device, it cannot completely offset the inherent potential inaccuracies compared to plasma glucose measurements. Importantly, for the formal diagnosis of diabetes, current guidelines recommend the measurement of fasting glucose in venous plasma after at least 8 h of fasting. Factors such as corticosteroid use, stress hyperglycemia, and nutritional status can impact glycemic control in oncology patients and their individual impact was not assessed in the included cohort. Some patients may have fasted for only a minimum of 4 h, which does not align with international guidelines for diagnosing diabetes. To address this, sensitivity analyses were conducted on patients in the early morning who were presumed to have fasted for at least 8 h to verify the findings.

In conclusion, non-optimal glycemic control is prevalent among melanoma patients regardless of diabetes diagnosis, potentially affecting both outcomes and quality of life. There remains an unmet clinical need and a lack of evidence-based guidelines for screening, assessing, and managing glycemic control in this patient group.

## CRediT authorship contribution statement

**Joan Walter:** Writing – original draft, Methodology, Funding acquisition, Formal analysis, Conceptualization. **Bojan Bojanic:** Writing – review & editing, Methodology, Data curation. **Manuel Dittli:** Writing – review & editing, Data curation. **Nadia Fehr:** Writing – review & editing, Data curation. **Thomas Sartoretti:** Writing – review & editing, Methodology, Data curation. **Moritz Schwyzer:** Writing – review & editing, Data curation. **Katharina Binz:** Writing – review & editing, Data curation. **Antonio G. Gennari:** Writing – review & editing, Methodology, Data curation. **Matthias Ernst:** Writing – review & editing, Data curation. **Martin W. Huellner:** Writing – review & editing, Data curation. **Michael Messerli:** Writing – original draft, Supervision, Methodology, Funding acquisition, Formal analysis, Conceptualization.

## Declaration of competing interest

The authors declare the following financial interests/personal relationships which may be considered as potential competing interests: Dr. Joan Walter and PD Dr. Michael Messerli are supported by a research grant from the Iten-Kohaut Foundation, Switzerland. Dr. Joan Walter reports research grants from the Swiss Heart Foundation, the Swiss Academy of Medical Sciences and Bangerter-Rhyner Foundation outside of the submitted work as well as consultant fees of Bayer and AstraZeneca.
